# Supplementary material for: Differential Effects of Two Isocaloric Healthy Diets on Postprandial Lipid Responses in Individuals with Type 2 Diabetes
Source: Nutrients. 2024 Jan 23;16(3):333. doi: 10.3390/nu16030333 (PMC10857261; doi:10.3390/nu16030333)

Supplementary Figure 2. Plasma glucose and insulin responses to a test meal evaluated as increase/decrease from fasting values (Panel A and C) and iAUC (Panel B and D) at baseline and after 8 weeks of dietary intervention with the MUFA (red lines and white bars) (n=22) or multifactorial diet (blue lines and bars) (n=21).
Data are mean ± SEM. *p <0.05 Multifactorial vs. MUFA diet, unpaired t-test; §p <0.05 Multifactorial vs. MUFA, 8-week values adjusted for baseline by ANCOVA.

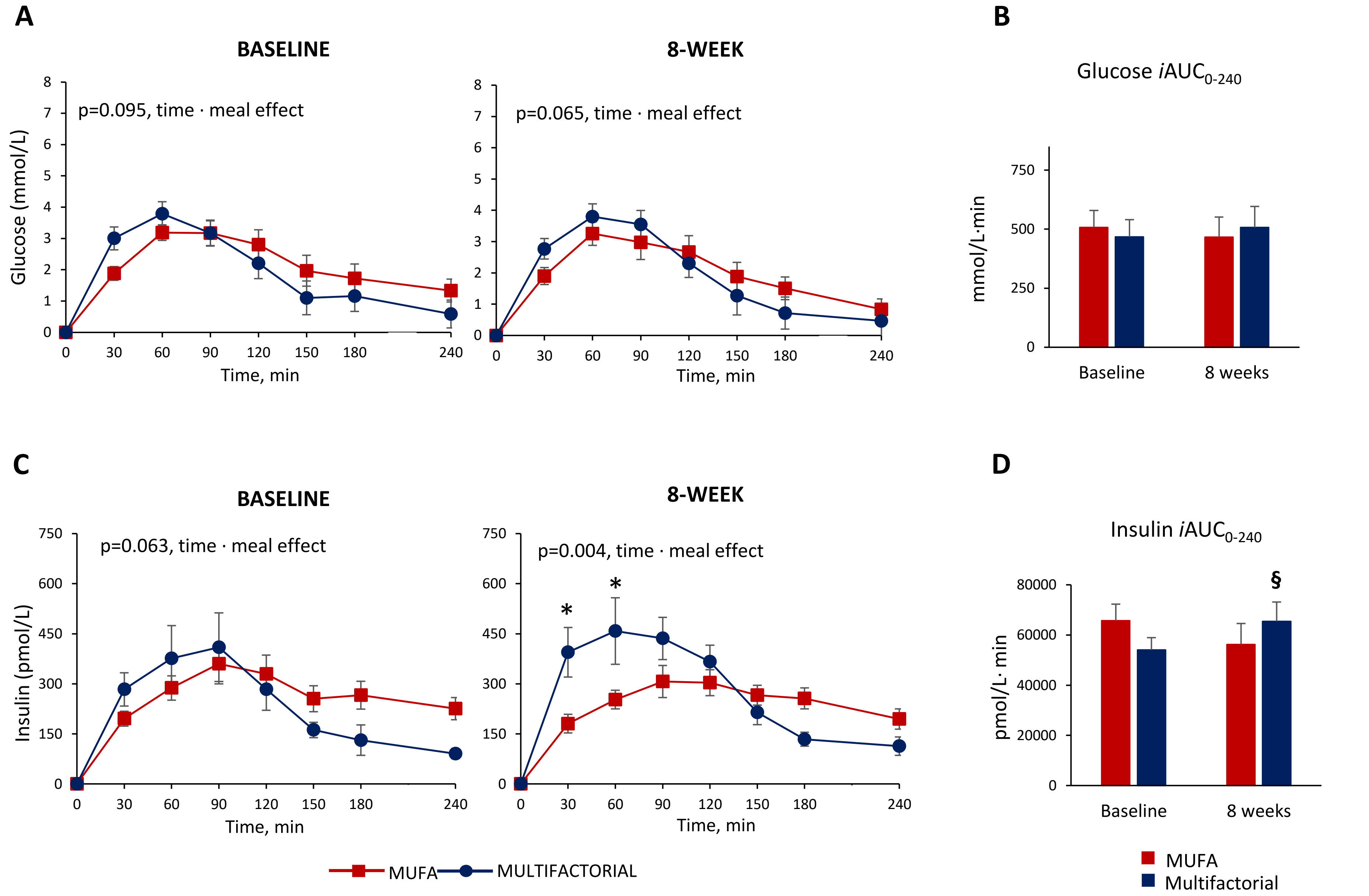

Supplement: Supplementary file 1 [file nutrients-16-00333-s001.zip › nutrients-2799058-supplementary/Supplementary Figure S2_Postprandial Glucose and Insulin Response.pdf]
