# Supplementary figures and images for: Differential Effects of Two Isocaloric Healthy Diets on Postprandial Lipid Responses in Individuals with Type 2 Diabetes
Source: Nutrients. 2024 Jan 23;16(3):333. doi: 10.3390/nu16030333 (PMC10857261; doi:10.3390/nu16030333)

Supplementary Figure 1. Flow Diagram

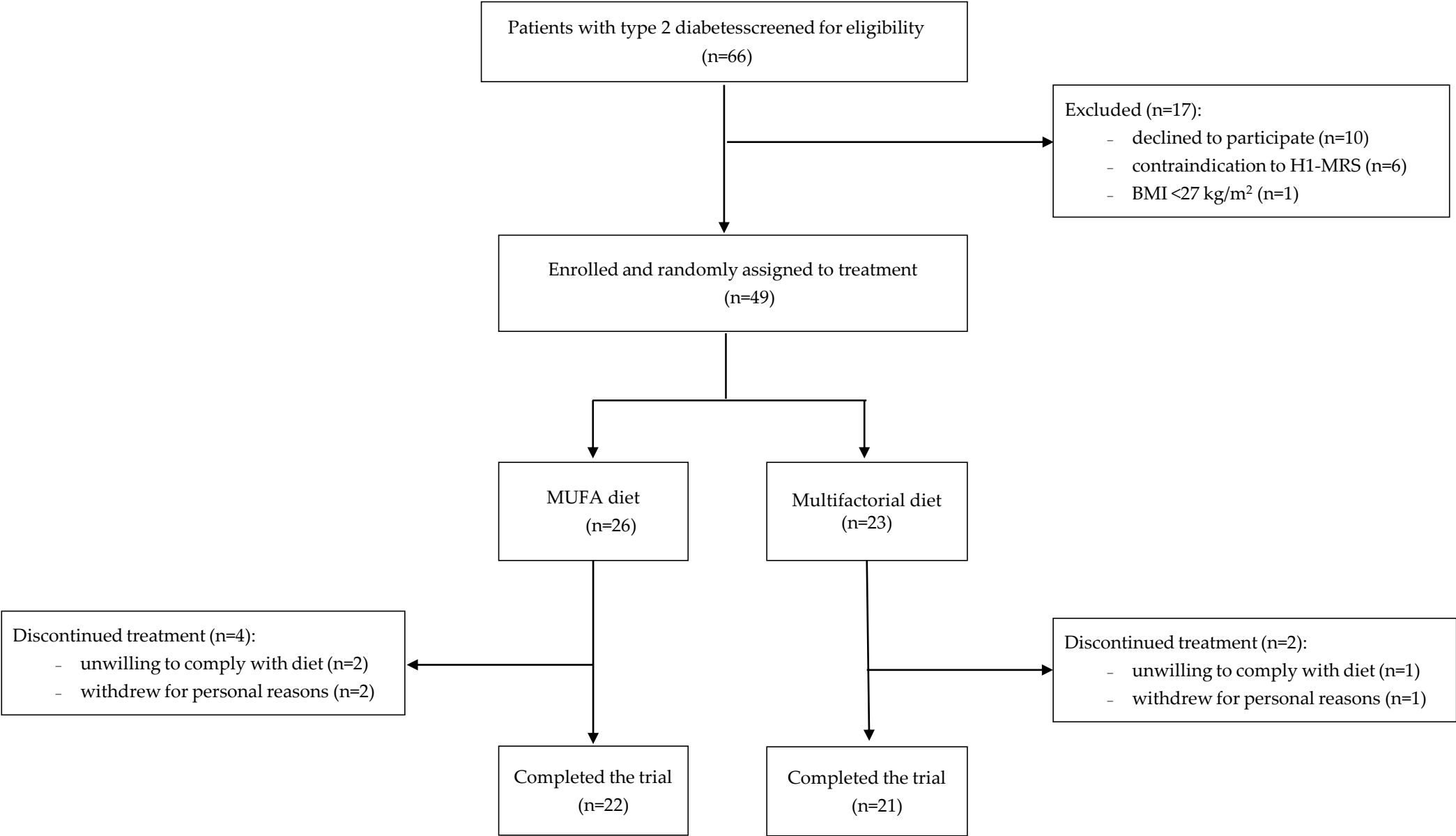

Supplement: Supplementary file 1 [file nutrients-16-00333-s001.zip › nutrients-2799058-supplementary/Supplementary Figure S1_ CONSORT FLOW DIAGRAM.pdf]
